# Supplementary material for: Dipole states and coherent interaction in surface-acoustic-wave coupled phononic resonators
Source: Nat Commun. 2019 Oct 8;10:4583. doi: 10.1038/s41467-019-12492-z (PMC6783409; doi:10.1038/s41467-019-12492-z)
Supplement: Supplementary file 1 — Supplementary Information [file 41467_2019_12492_MOESM1_ESM.pdf]

Supporting information
Dipole states and coherent interaction in surface-acoustic-wave coupled mechanical resonators

Raguin *et al.*
Institut FEMTO-ST, CNRS, Université de Bourgogne Franche-Comté
15B Avenue des Montboucons, F-25030 Besançon Cedex, France and
Institut FEMTO-ST, CNRS, Université de Bourgogne Franche-Comté
15B Avenue des Montboucons, F-25030 Besançon Cedex, France

I. SAMPLE FABRICATION

Interdigital transducers

Interdigital transducers were fabricated by photolithography followed by a 200-nm thick Al layer deposition and subsequent lift-off on a 500 µm-thick lithium niobate substrate ($LiNbO_3$) in the Y-crystallographic orientation. The transducers were designed with a chirped finger period in order to operate in a frequency range between 50 and 100 MHz, which covers the frequency range of the first flexural mode of the designed resonators.

Microresonators

The $LiNbO_3$ substrate was installed in a FEI Helios Nanolab 600i Dualbeam FIB/SEM system equipped with five different gas injection systems (GIS). The GIS dedicated to platinum deposition is used to locally inject the trimethyl-(methylcyclopentadienyl)-platinum(IV) precursor on the sample. The position of the needle in lateral and height distances from the coincidence point of the two beams was optimized to reach a compromise between growth speed and dimensional uniformity of the structures in the considered write field of 50 µm. After opening the GIS valve, a stabilisation time of two minutes was set to homogenize the vacuum chamber parameters. The working pressure reached is 1.5×10^{-5} mbar.
The pillars were grown with an accelerating voltage of 30 kV and a probe current of 40 pA. The beam is scanned repetitively and at a high speed on the growth area to optimize the refreshment of the precursor. The growth parameters are as follows: stepsize of 40 nm and dwelltime of 200 ns with a number of loops depending of the desired height and ranging from 1.5×10^6 to 2.3×10^6. The probe current is indeed low (3.18 $pA.cm^{-2}$ for a pillar of 4 µm in diameter) but was chosen carefully to limit the roughness of the structures on the sidewalls and on the top surface. The exposure dose is constant and equal to 0.4 $\mu m^3.nC^{-1}$. The atomic composition of the deposited material was characterized by Energy-dispersive X-ray spectroscopy (EDS) leading to a ratio of 29 at.% of platinum, 18 at.% of gallium and 53 at.% of carbon. The estimated Young Modulus and mass density are of the order of 130 GPa and 1×10^4 $kg.m^{-3}$ respectively.

Resonators within a pair were grown simultaneously: the ion beam scans alternatively, at a speed of 200 μ $m.s^{-1}$, the two growth area. This process results in the production of twin pillars. In order to improve further the planeity of the top surface and to gain a higher degree of control on the pillar height, their upper faces were polished by ion beam milling. A dedicated substrate holder was designed and manufactured to bring the pillar orthogonal to the ion beam. The probe current used for this step is 430 pA. Although both pillars within a pair were processed within a single milling step, a difference in height of a few tens of nanometers between the two pillars may remain, which could result in a potential difference in the resonance frequency of the first flexural mode (approximately 3 MHz for a difference in height of 100 nm).

II. OPTICAL CHARACTERISATIONS

A laser scanning heterodyne interferometer was used to characterize the resonator behaviour by measuring the out-of-plane displacement field distribution and phase maps. The set-up is a heterodyne Mach-Zehnder interferometer, much similar to the one proposed in [1] and displayed in Figure 1a.

A linearly polarized, stabilized 633-nm HeNe laser (Thorlabs HRS015B, nominal output power 1.2 mW) is split into two frequency-shifted paths thanks to an acousto-optic modulator chosen for the large separation angle between the two diffraction orders (AA Opto Electronic MTS110-A3-VIS) after passing through an optical isolator. The first-order, frequency-shifted beam acts as the reference arm while the zeroth-order beam propagates to the sample and back. The zeroth- and first-order beams exhibits orthogonal polarisations.

The reference beam is directly transmitted through the polarizing beamsplitter. The probe beam propagates through a half-wave plate, passes through the polarizing beamsplitter and is sent through a quarter-wave plate before reaching the sample. The laser beam is focused on the sample using a long-working distance microscope objective with a numerical aperture of 0.8 (Olympus LMPLFLN100x), leading to a spot size of 650 nm. After reflection on the sample, the second pass through the quarter-wave plate allows to rotate the beam polarisation so that the probe beam is now reflected on the beam-splitter. This way, the two orthogonally-polarized beams are recombined. The resulting interference pattern allows to calculate the optical phase difference between the two paths and to determine the amplitude of the out-of-plane component of the sample surface vibration. The two interfering beams propagate through a linear polarizer to a fast photodiode (Alphalas UPD-200-SP). The signal collected by the photodiode is amplified and then either sent to an electrical spectrum analyzer for

amplitude measurements (Anritsu MS2830A) or to an oscilloscope (Agilent DSO9254A) to perform a numerical modulation of the signal and extract the corresponding phase maps.

The frequency responses and field and phase maps are then retrieved by driving the resonators at a single-frequency within the IDT operating range using a signal synthesizer (Agilent N5181A). For all reported measurements, the radio-frequency drive power for the IDTs was set at 20 dBm.

The laser beam can be focused either on the pillar surface or on the substrate surface. The low depth of field of the optical set-up in conjunction with the pillar height allows us to probe the displacement field at these two locations in an independent fashion.

The field maps are obtained by raster scanning the surface of interest for specific frequencies. In this case, the scans covering the area of interest are realized with a 200-nm or 500-nm step size. As depicted in Figure 1b, the frequency response of each resonator is retrieved by measuring the out-of-plane vibration amplitude on 16 points describing a circle with a diameter about equal to one half of the cylindrical resonator diameter and by recording the maximum amplitude value out of these 16 points for each excitation frequency. The circle diameter is chosen so that the laser spot remains focused on the pillar surface at all times. This method allows to record frequency responses with an accurate frequency step (10 kHz) adapted to the frequency behaviour of the pillar pairs over a large frequency domain (up to 10 MHz), in a tolerable time. This measurement principle is particularly well suited to the nature of the considered vibration mode of the pillars: the fundamental flexural mode indeed implies that the out-of-plane displacement varies linearly along the radius of the pillar surface orthogonal to the nodal line. Then, by measuring the out-of-plane displacement on a precise point of the surface, it is possible to deduce the maximum out-of-plane displacement (at the edge of the pillar surface), knowing the position of the measured points and the pillar diameter. The chosen number of points in addition allows to retrieve the position of the nodal line and hence the orientation of the mode vibration. In all cases, the scanning of the sample is accomplished using computer-controlled nanometer precision linear positioners (SmarAct SLC-1760-s).

The vectorial nature of the resonator motion, i.e. their amplitude, phase, and eigenmode orientation, can therefore be characterized with submicron precison, provided that the considered eigenmode displacement fields exhibit a non-zero out-of-plane polarisation component.

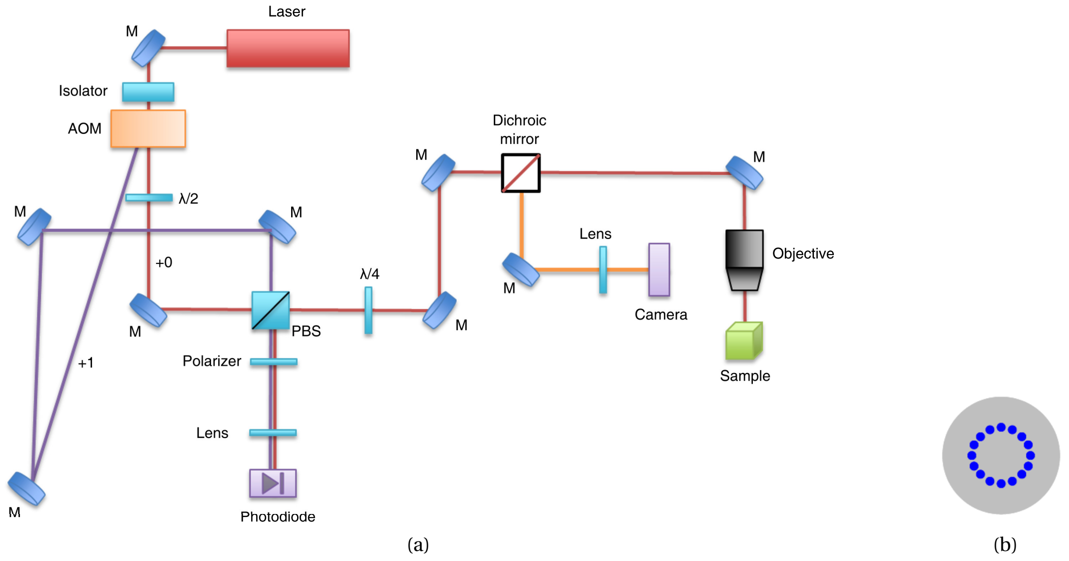

Figure 1. (a) Schematic of the experimental set-up used for optical characterisations. (AOM = Acousto-Optic Modulator; PBS = Polarized Beam Splitter). (b) Principle of the 16-point based resonance frequency measurement method.

III. DATA PROCESSING

The straight lines accompanying the raw data sets provided on the frequency scans were obtained by performing a locally weighted linear regression on the data upper peak envelope, after removal of outliers, as detailed further below. We chose not to perform a simple average of the data after analysis of the response of the chirped interdigital transducers used to excite the SAW. The IDT design has indeed not been optimized enough to prevent internal reflections within the electrodes,

which is a very difficult issue in such a broadband regime. These internal reflections result in destructive interference, hence canceling some output frequencies. An example of such frequency response is reported in Figure 2.

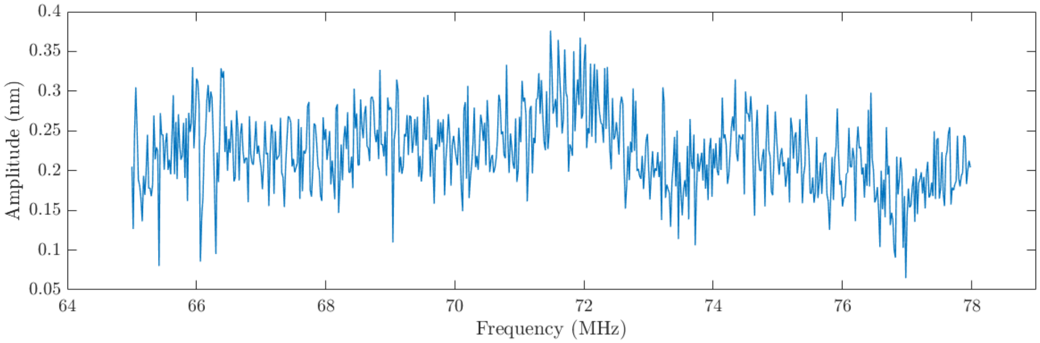

Figure 2. Typical experimental frequency response of an interdigitated transducer measured with a 20-kHz step in a frequency band ranging from 65 to 78 MHz.

This interrupted emission obviously has an impact on the resonator excitation, as a resonator cannot be excited if no SAW is generated by the transducer. The impact of this effect, and the deterministic character of these extinctions are shown in Figure 3. Here, the out-of-plane amplitude of a single point taken at the surface of a pillar excited by a SAW is reported. The measurement has been repeated 8 times. The plot represents the mean amplitude for each frequency point, the error bar represent the 95% confidence level. The measurements show a good overlap of the amplitude data points, therefore ruling out interpreting the observed fluctuations as additive Gaussian noise.

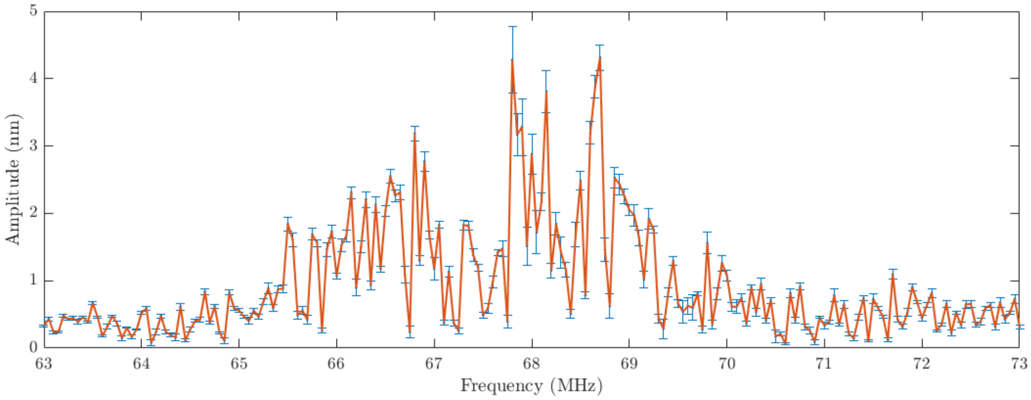

Figure 3. Single point measurement performed on a resonator excited by a SAW. The laser probe was focused on a point located at the pillar edge, the position was kept fixed when performing the frequency scan from 63 to 73 MHz. The measurement was repeated 8 times over the frequency range of interest. The error bars represent the 95% confidence level.

Using a moving average filter would therefore result in including the zero-amplitude points linked to the absence of incoming surface wave, resulting in a significant underestimation of the amplitude. We therefore chose to work on the envelope of the signal, and adopted the following method. The local maxima of the measured signal were searched within successive moving windows. The window width was set to 230 kHz. The obtained maxima were then sorted and the 10% upper values considered as outliers and removed from the data set. A locally weighted non-parametric regression fitting using a 2nd-order polynomial was then applied on the data upper peak envelope to smooth the data.

IV. NUMERICAL MODEL

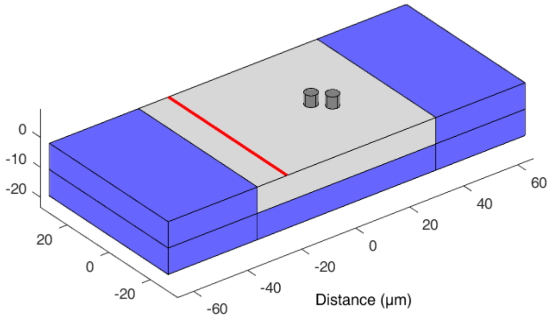

Figure 4. Schematic of a structure used for the numerical simulations. Here, we compute 1.5 μm-spaced pillars in -45°-configuration. The source is depicted in red. The domains colored in blue correspond to the PML.

The numerical simulations were performed using the Finite Element Method (FEM), through the Comsol Multiphysics software (V3.4). We implement time harmonic simulations thanks to the Partial Differential Equations Module using a formulation taking into account the anisotropy and piezoelectricity of the lithium niobate substrate. The material constants of lithium niobate are taken from [2]. The resonators are considered being made of an isotropic material with a Young's modulus of 130 GPa and a mass density of 1×10^4 kg.m^{-3}, as in Ref [3]. The implemented equation is then:

$$-\rho\omega^2 u_j = \frac{\partial}{\partial x_j}\left(c_{ijkl}\frac{\partial u_l}{\partial x_k} + e_{kij}\frac{\partial \phi}{\partial x_k}\right), \quad (1)$$

where i, j, k are integers between 1 and 3, ρ corresponds to the density of the considered material, ω is the pulsation of the excitation, u_j is the displacement along direction j, c_{ijkl} are the elastic constants, e_{kij} the piezoelectric constants and ϕ depends on the electric field as follows: $\mathrm{E}_k = -\frac{\partial \phi}{\partial x_k}$.

The simulation domain is depicted in Figure 4, it consists in a rectangular structure with dimensions superior to the studied wavelengths (66 μm long and 58 μm large). A line source is used as a frequency excitation: a mechanical displacement with sagittal polarisation and 1-nm out-of-plane amplitude is applied along the line to generate the traveling wave. To avoid backward reflections, perfectly matched layers (PML) are added at the bottom of the propagation domain and at the two boundaries of the wave propagation axis. On the line source axis, we employ periodic boundary conditions in order to avoid disturbing the straight wavefront, taking care to keep a sufficient distance between two periods to avoid coupling between two successive pairs of pillars (here, the distance was set at 58 μm). Free boundary conditions were applied on the surface of the substrate and of the deposited pillars, all other boundaries are clamped.

In order to mimic the experimental conditions, we assume a difference in height of 100 nm between two pillars within a pair. The pillar diameters was therefore set at 4.4 μm and the height at 4.0 and 3.9 μm respectively. Frequency responses for each pillar were obtained by extracting the maximum amplitude on the pillar surface.

V. SUPPLEMENTARY EXPERIMENTAL DATA

Amplitude and phase measurements were performed on similar pairs of pillars (nominal diameter of 4.4 µm and nominal height of 4 µm, gap distances of either 1.5 or 6 µm) and are presented here as supportive information for Figure 3 and Figure 4 of the main manuscript.

Longitudinal excitation, 1.5-µm gap distance

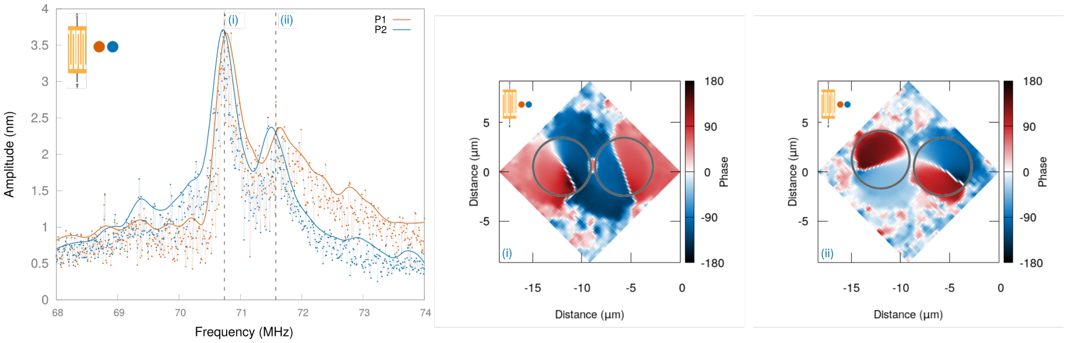

Figure 5. Experimental frequency responses of 1.5-µm-spaced pillars in the longitudinal-excitation configuration. Phase maps are given for frequencies f equal to 70.74 MHz (i) and 71.58 MHz (ii), corresponding to the two modes of the resonator pair. The scan area is 13 µm×13 µm, with a step size of 200 nm.

Figure 5 shows the frequency response obtained in the case of a longitudinal excitation scheme, for a different set of coupled pillars fabricated, excited and characterized in the same conditions as the one presented in Figure 3 of the main article. If the overall frequency response behaviours are similar, the modes splitting is clearer in the case of this particular sample, which is probably due to reduced fabrication imperfections in this subsequent series of fabricated resonators. Amplitude and phase measurements however confirm that the modal behaviour is conserved. Figure 5 indeed displays phase measurements taken at frequencies of 70.74 MHz and 71.58 MHz. Each of these frequencies correspond to a mode of the dimer and can be compared to frequencies labeled (i) and (ii) in Figure 3 of the main manuscript. The orientation of the pillars is comparable, as shown by the position of the nodal line. For both modes, the two resonators vibrate out-of-phase. The observed behaviour confirms that these two modes can be identified with modes (ii) and (iii) of the case of a diagonal incidence (Figure 2 of the main manuscript).

Note that the plots have been rotated by 135° to account for both a rotation of the IDT source and pillar pair on the substrate surface and for a rotation of the sample with respect to the X and Y directions of the substrate holder. The position of the source with respect to the pillar pair is shown as an inset.

Transverse excitation, 1.5-µm gap distance

The pillar pairs subjected to a transverse excitation considered in the main part of the manuscript exhibited unstable polarisation states that led to quasi-circular polarisation states of motion. These unstable states are characteristics of the sensitivity of the proposed coupling scheme to the geometrical features of the resonators. Figure 6 reports the frequency response obtained for an other pair of pillars, fabricated and characterized along the same conditions. The frequency response is overall similar to the one reported in Figure 4a of the main manuscript, although the mode splitting for each resonator is more clearly resolved. The maxima of vibration amplitude are reached at almost the same frequencies for the two resonators. Mode (i) appears at about 67 MHz while mode (ii) is found at about 71 MHz for both resonators. Experimental field maps show that this particular sample exhibits more stable polarisation states, leading to well-defined and well-oriented flexural modes. This allows proceeding to an easier identification of the modes selected by the source with respect to the more general diagonal incidence case. Phase field maps taken at $f = 67.25$ MHz for mode (i) and at $f = 70.86$ MHz for mode (ii) show that the nodal line is here clearly defined. Mode (i) corresponds to a well-aligned symmetrical state where the resonators vibrate in-phase, as observed for mode (i) in the case of a diagonal incidence. Mode (ii) is anti-symmetrical and can be identified to mode (iii) of the diagonal case. The symmetrical mode with the pillar vibration oriented orthogonally to the inter-resonator axis is then ruled out by the transverse excitation scheme.

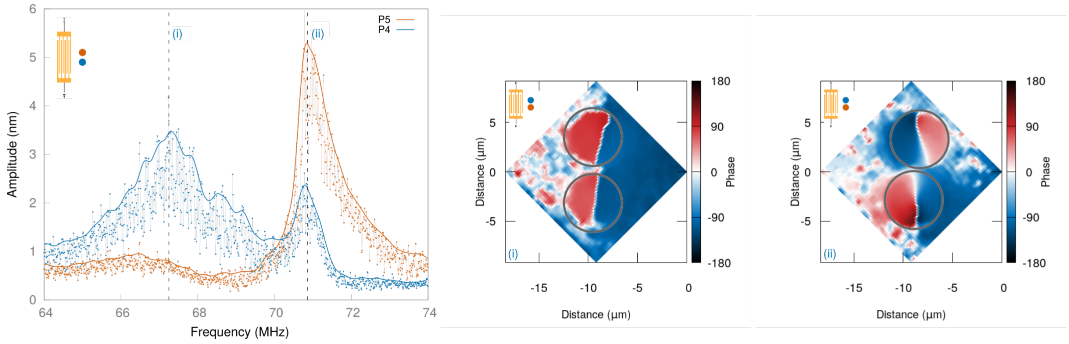

Figure 6. Experimental frequency responses of 1.5-µm-spaced pillars in the transverse-excitation configuration. Phase maps are given for frequencies f equal to 67.25 MHz (i) and 70.86 MHz (ii), corresponding to the two modes of the resonator pair. The scan area is 13 µm×13 µm, with a step size of 200 nm.

Again, the plots have been rotated by 135° to account for both a rotation of the IDT source and pillar pair on the substrate surface and for a rotation of the sample with respect to the X and Y directions of the substrate holder. The position of the source with respect to the pillar pair is shown as an inset.

Longitudinal excitation, 6-µm gap distance

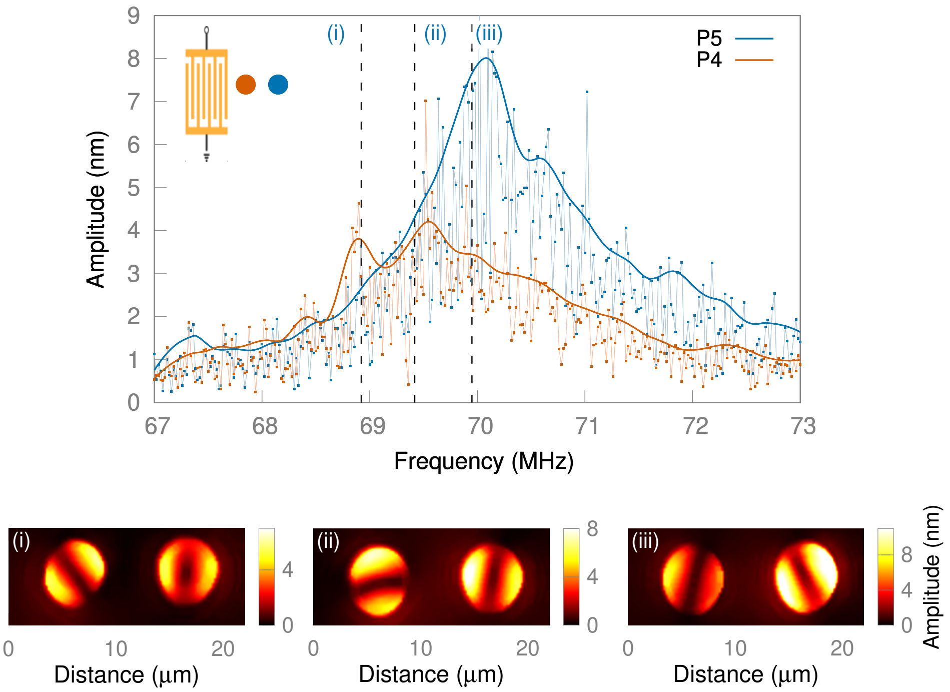

Figure 7. Experimental frequency responses of 6 µm-spaced pillars in the longitudinal-excitation configuration. Bottom: corresponding out-of-plane displacement field maps for typical excitation frequencies: 68.92 MHz (i), 69.42 MHz (ii), and 69.95 MHz (iii)). The scan area is 22 µm×7 µm, with a step size of 200 nm.

Additonal pillar pairs with gap distances of 6 µm were also fabricated to confirm the presence of orthogonal polarisation states for increased gap distances, regardless of the incident surface acoustic wave vector direction. We now focus on a longitudinal excitation scheme. Figure 7 reports the obtained frequency responses. Here, the frequency response overlap is higher than the one reported in Figure 4b of the main manuscript. Again, this is most probably due to the variability of

the fabrication process that has most probably yielded here resonators with a weaker height difference (again, a difference in pillar height of 100 nm results in a frequency shift greater than 2 MHz for an individual pillar). The observed resonances are closer to the one exhibited by a single resonator. The frequency response is however broadened, as observed in the case of the transverse excitation reported in Figure 4b of the article. Additionally, pillar P4, located closest to the source, seems to exhibit two distinct resonances, pointing at resonator-to-resonator coupling. The amplitude field maps reported in Figure 7 confirm the occurrence of orthogonal polarisation states at frequencies close to the crossing point, here shown for $f = 69.42$ MHz. Again, these orthogonal polarisations states are expected in the case of a mechanical coupling but not in the context of a dipole-dipole interaction.

VI. SUPPLEMENTARY REFERENCES

[1] K. Kokkonen and M. Kaivola, “Scanning heterodyne laser interferometer for phase-sensitive absolute-amplitude measurements of surface vibrations”, Appl. Phys. Lett. **92**, 63502 (2008).

[2] G. Kovacs, M. Anhorn, H. E. Engan, G. Visintini, and C. C. W. Ruppel, in “IEEE Ultrason. Symp. Proc.” (1990), vol. 1, pp. 435–438.

[3] S. Benchabane, R. Salut, O. Gaiffe, V. Soumann, M. Addouche, V. Laude, and A. Khelif, “Surface-Wave Coupling to Single Phononic Subwavelength Resonators”, Phys. Rev. Appl. **8**, 034016 (2017).
